# Windows to Consciousness: The Role of Fronto-Parietal Connectivity in Anesthesia-Induced Unconsciousness

**DOI:** 10.2174/011570159X375644250405041050

**Published:** 2025-05-15

**Authors:** Yuanyuan Ding, Shiya Liu, Kaixin Wang, Junya Kang, Wenqi Chen, Shujun Sun, Yuxin Hu, Yunyun Han, Xiangdong Chen

**Affiliations:** 1 Department of Anesthesiology, Union Hospital, Tongji Medical College, Huazhong University of Science and Technology, Wuhan, 430022, Hubei Province, China;; 2 Institute of Anesthesia and Critical Care Medicine, Union Hospital, Tongji Medical College, Huazhong University of Science and Technology, Wuhan, 430022, China;; 3 Key Laboratory of Anesthesiology and Resuscitation (Huazhong University of Science and Technology), Ministry of Education, Wuhan, China;; 4 Department of Neurobiology, School of Basic Medicine, Tongji Medical College, Huazhong University of Science and Technology, Wuhan, 430022, China

**Keywords:** Consciousness, anesthesia, fronto-parietal connection, information integration, neuroimaging studies, loss of responsiveness

## Abstract

The exploration of consciousness and the elucidation of the mechanisms underlying general anesthesia are two intertwined endeavors that have significantly advanced our understanding of the neural correlates of awareness. Both fields converge on the neural systems that regulate consciousness. Frontoparietal networks, known for their involvement in executive functions, attention, and cognitive control, emerge as key players in the transition from wakefulness to anesthesia-induced unconsciousness. This review synthesizes recent findings highlighting the pivotal role of fronto-parietal connectivity in the induction and maintenance of unconsciousness by general anesthetics. By examining functional neuroimaging studies and neurophysiological data, we elucidate how disruptions in fronto-parietal interactions contribute to the loss of responsiveness and altered states of awareness associated with anesthesia. Additionally, we further explain the underlying mechanism at both the neuronal and molecular levels. Furthermore, we discuss the implications of these findings for advancing our understanding of the neural correlates of consciousness and the development of novel anesthetic agents with more predictable and targeted effects on consciousness. This review decisively bridges the gap between consciousness research and anesthetic pharmacology, providing a robust framework for future investigations into the neural mechanisms that control transitions between conscious states.

## 1 INTRODUCTION

General anesthesia, which produces a reversible loss of consciousness, has a pivotal role in painless surgery in medical practice. The Lancet reported that over 300 million patients worldwide receive general anesthesia every year [1]. Despite the shared effect of inducing loss of consciousness, general anesthetics vary significantly in their chemical structures and targetsHow various anesthetic agents induce similar states of unconsciousness is a topic of mutual interest for anesthesiologists and neurobiologists. Studying the neuroscientific basis of anesthesia not only enhances our comprehension of its mechanisms but also provides valuable insights into the nature of intangible consciousness and cognition [2-4]. Moreover, understanding general anesthesia mechanisms aids anesthesiologists in achieving personalized anesthesia for patients and designing safer anesthetic agents.

The theory of the anesthesia mechanism has evolved through several stages. From the “lipid theory” to the “protein target theory” and currently to the “neural circuit regulation theory” [5-9], we gradually deepen our knowledge of the mechanism of action of general anesthetics. The last decade has also seen a surge in breakthroughs in the quest for underlying mechanisms driven by the development of experimental technologies [10-14]. It has long been established that the cerebral cortex is a primary target of anesthetics. Recent studies, such as those conducted by Krom *et al*. (2020) [15], Bharioke *et al*. (2022) [16], and Esien *et al*. (2024) [17], highlight that general anesthetics modulate dynamic connectivity within the cerebral cortex, particularly influencing the communication between the frontal cortex and the parietal cortex [7, 18, 19]. The precise mechanisms by which anesthetics affect consciousness remain unclear, while there is increasing evidence suggesting that disruption of fronto-parietal communication correlates with loss of consciousness caused by anesthetic agents. This review focuses on discussing how alterations in fronto-parietal connectivity contribute to unconsciousness under anesthesia and elucidating the mechanisms by which anesthetic agents disrupt this connectivity.

In this review, we will first provide a brief overview of the anatomy of the cerebral cortex and the function of fronto-parietal cortical connectivity. Then, we discuss the effects of various anesthetics on fronto-parietal cortical connectivity from fMRI and EEG evidence. Finally, we will explore the mechanisms underlying the disruption of fronto-parietal cortical connectivity by anesthetics from neural circuits and cellular neuroscience perspectives.

## 2 ANATOMY AND FUNCTION OF FRONTO-PARIETAL CORTICAL CONNECTIVITY

**Fig. (1):**
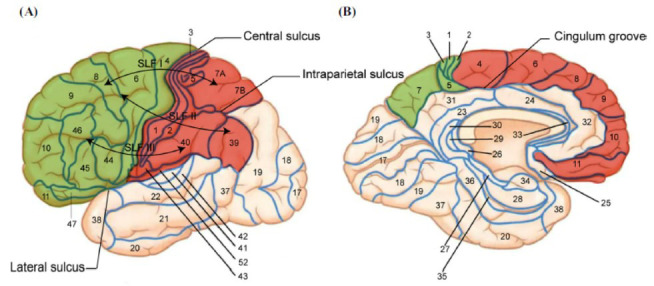
Diagram of the main frontal and parietal regions in Brodmann’s map together with their association pathways. (**A**) Lateral view. The frontal lobe (green area) lies above the lateral sulcus, separated from the parietal lobe (red area) by the central sulcus. The parietal lobe transforms caudally into the occipital lobe without a definite landmark, it is split into the superior parietal lobule (SPL) and the inferior parietal lobule (IPL) by the intraparietal sulcus (IPS). The superior longitudinal fascicle is schematically drawn, which is the major long association pathway between the frontal lobe and the parietal lobe. (**B**) Mesial view. On the medial surface, both the frontal lobe and parietal lobe are located above the cingulum groove.

In clinical settings, surface anatomy retains significant value as it serves as the foundation for understanding brain functions and behaviors, particularly in supporting functional connectivity networks [20]. The central sulcus separates the frontal lobe from the parietal lobe, defining their distinct functions in the brain. The frontal lobe is located anterior to the central sulcus and above the lateral sulcus (Fig. **1**) [21]. The parietal lobe extends over the lateral and medial surface of the brain, from the lateral sulcus ventrally to the cingulate groove medially (Fig. **1**) [22, 23]. Korbinian Brodmann subdivided the cerebral cortex into 52 areas based on functional and pathological criteria [24]. In Brodmann’s map, areas 4, 6, 8, 9, 10, 11, 12, 25, 33, 44, 45, 46, and 47 belong to the frontal cortex, while the parietal cortex includes areas 1, 2, 3 5, 7, 39, 40, and 43 [25-28].

As the general principle of neural network, white matter fiber pathways connect neocortical areas and with subcortical structures, serving as substrates for cognition [29]. Regarding the linkage between the parietal lobe and the frontal lobe, neighborhood association fibers of the anterior parietal cortex are focused on the close vicinity of the frontal areas around the central sulcus, while the posterior parietal cortex connects with the frontal cortex through a long association fiber tract named superior longitudinal fasciculus (SLF) [30]. These tract tracing observations are supported by diffusion tensor imaging (DTI), probabilistic tractography, and functional connectivity mapping in human subjects. The SLF has three subcomponents (I-III): SLF I links the superior parietal region and adjacent medial parietal cortex with the frontal lobe’s supplementary and premotor areas. It is believed to regulate higher aspects of motor behavior requiring positional information about body parts and may contribute to the initiation of motor activity; SLF II links the caudal inferior parietal lobule and parieto-occipital areas with the posterior part of the dorsolateral and mid-dorsolateral prefrontal cortex. This facilitates visual awareness, maintenance of attention, and environmental engagement, allowing the prefrontal cortex to regulate attention within different parts of space; SLF III connects the inferior parietal and adjacent intraparietal sulcus regions with the ventrolateral premotor and prefrontal cortices. It offers higher-order somatosensory input to the ventral premotor region and pars opercularis, thus playing a crucial role in orofacial and hand actions, as well as phonemic and articulatory aspects of language [31, 32]. These fiber tracts, with their characteristic functional properties, are the basic anatomical substrates for information exchange between the parietal and frontal regions.

## 3 EFFECTS OF ANESTHETIC AGENTS ON FRONTO-PARIETAL CORTICAL CONNECTIVITY FUNCTIONAL CONNECTIVITY AND INFORMATION TRANSFER

In recent years, there has been a proliferation of theories concerning the neural basis of consciousness. Four prominent theoretical models—higher-order theory, global workspace theory, integrated information theory, and re-entry and predictive processing theory—have garnered significant scholarly attention [33]. (1) Higher-Order Theory: Some higher-order theories emphasize the importance of prefrontal regions, especially the prefrontal cortex, due to their association with complex cognitive functions and higher-order representations related to consciousness. However, not all higher-order theories focus solely on these regions for the maintenance of consciousness. (2) Global Workspace Theory: The theory emphasizes the broadcasting of information within a large-scale neuronal workspace as being related to consciousness, with the frontal-parietal cortical regions playing a hub-like role. According to this theory, these regions are critical for both the generation and maintenance of consciousness. (3) Integrated Information Theory (IIT): Unlike the previous two theories, IIT links consciousness primarily with posterior cortical areas (referred to as “posterior hot zones”, including parietal, temporal, and occipital regions), believing that these regions' neuroanatomical properties are suited to generate high levels of integrated information. It does not emphasize the crucial role of frontal and parietal regions in maintaining consciousness. (4) Re-entry and Predictive Processing Theory: In the re-entry theory, such as the local recursive theory, local recursive or re-entry processing within perceptual cortices is sufficient to generate consciousness. Frontal and parietal regions may be necessary for reporting perceptual experience content or for reasoning and decision-making, but their core role in maintaining consciousness is not emphasized. The predictive processing theory typically explains local conscious states in terms of top-down perceptual predictions, without stressing the frontal and parietal regions’ critical role in maintaining consciousness, although it does address the functions of these regions concerning consciousness and attention [34-36].

Some studies have shown the fronto-parietal connection is pivotal for integrating information across different sensory and cognitive domains to form a unified and coherent conscious experience [34, 37-42]. Specifically, neuronal groups in the prefrontal cortex are responsible for executive functions such as goal setting, planning, and working memory, while the parietal lobe is involved in spatial information processing, attention allocation, and sensorimotor integration [18, 43]. Effective connectivity between these two brain areas facilitates the coordination and integration of information across different cognitive domains, thereby supporting the state of consciousness. In cortical areas, feedforward pathways transmit sensory information from the lower-order cortex (parietal cortex) to the higher-order cortex (frontal cortex), which in turn sends feedback to modulate information selection in the lower-order cortex [44]. This interaction is dynamic, adapting to task demands and environmental changes. For instance, when performing a task requiring planning and attention, the prefrontal cortex directs parietal attention to relevant sensory information, while feedback from the parietal cortex enables the prefrontal cortex to refine its action plans [19, 45]. Previous studies have revealed that the frontal cortex could be a hub that integrates the level and content of consciousness [19], while the parietal cortex is considered a “hot spot” for the neural correlates of consciousness [38, 46]. Thus, this bi-directional processing is believed to be a key factor in the creation of consciousness, and impaired function or disrupted connectivity in fronto-parietal regions compromises the integrity of consciousness.

## 4 ELECTRICAL ACTIVITY UNDER DIFFERENT ANESTHETIC-INDUCED UNCONSCIOUSNESS

**Fig. (2):**
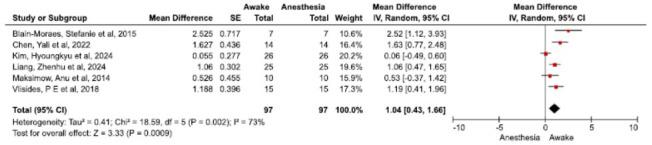
Meta-analysis by anterior-posterior connectivity during awake or anesthesia.

Systems neuroscience research has reported that general anesthesia—produced by agents such as propofol, sevoflurane, and ketamine—suppressed fronto-parietal connectivity, despite differing molecular targets [38, 47-54]. However, there is no clear evidence to demonstrate this result. Therefore, we aimed to review the literature to evaluate the effect of anesthetics on fronto-parietal connectivity. We searched 4 electronic databases (PubMed, Embase, Web of Science, and Cochrane) using a combination of free text and medical subject headings terms related to “propofol”, “sevoflurane”, “isoflurane”, “ketamine”, “anesthesia”, “anesthetic”, “consciousness”, “electroencephalogram”, and “functional magnetic resonance imaging” from September 2014 to September 2024. A search was limited to English-language articles. After excluding identical literature, we selected articles meeting the following criteria. Inclusion criteria: (1) Studies (fMRI or EEG) evaluating the effect of anesthetics on fronto-parietal connectivity in humans/primates were reviewed. (2) All subjects in the selected studies had no brain disease. (3) Studies have primary data. (4) Control conditions (*e.g*., wakefulness) were reported. Exclusion criteria: (1) Letters to the editor, single case reports, reviews, or abstracts were excluded. (2) Non-peer reviewed articles were excluded. (3) Non-pharmacological unconsciousness (*e.g*., coma, sleep) was excluded. Pairs of authors independently screened the studies and extracted data. Discrepancies were resolved through discussion with a third investigator. Meta-analyses were performed with RevMan (version 5.3). Table **1** represents key functional magnetic resonance imaging (fMRI) and electrophysiological evidence demonstrating alterations in fronto-parietal connectivity during general anesthesia. During anesthesia-induced unconsciousness, the prefrontal cortex is frequently observed to separate from the posterior cortex, a phenomenon that occurs with nearly all general anesthetics (Table **1**). We performed a random-effects meta-analysis of the data presented in Table **1**, excluding studies that lacked raw data (Fig. **2**). Meta-analytic pooling of the anterior-posterior connectivity during awake or anesthesia reported by 6 studies yielded a crude mean difference (MD) across studies was 1.04 (97 participants; 95% CI, 0.43-1.66), indicating a significant overall effect of anesthesia on anterior-posterior connectivity (Z = 3.33, *P* = 0.0009). Substantial heterogeneity was observed among the studies (I^2^ = 73%, *P* = 0.002), suggesting variability in the effect sizes across different studies. This heterogeneity may be attributed to differences in study designs, anesthesia protocols, or measurement techniques. The meta-analysis demonstrates that anesthesia significantly decreases anterior-posterior connectivity. The disruption of connectivity along the anterior-posterior axis, following the administration of an appropriate anesthetic dose, coincides with a diminished transfer of information along this axis—affecting both feedforward and feedback pathways [38, 50, 53]. This disruption is linked to the onset of unconsciousness. However, the question of whether the loss of such connectivity is both necessary and sufficient for inducing unconsciousness remains unresolved. It is hoped that advancements in appropriate assays and vibrant definitions of consciousness will ultimately provide answers in the future.

**Fig. (3):**
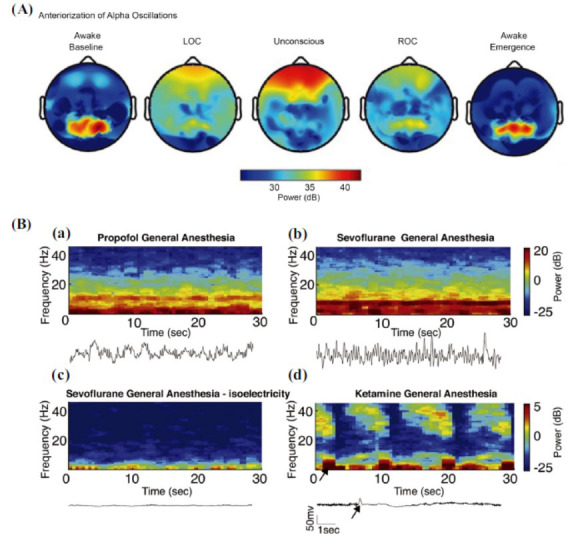
Electroencephalography (EEG) features of different anesthetics. (**A**) Anteriorization of alpha oscillations. (**B**) EEG patterns of several common anesthetics. (**A**) adapted from Purdon *et al*. [59], (**B**) adapted from Akeju and Brown [106]. (**a**) EEG signatures of propofol-induced general anesthesia, (**b**) EEG signatures of sevoflurane-induced general anesthesia, (**c**) Isoelectric EEG activity during deep sevoflurane anesthesia, (**d**) EEG signatures of ketamine-induced general anesthesia.

Electroencephalography (EEG) is a powerful and non-invasive tool for probing the synchronized firing of neuronal populations in the cortex [55, 56]. Owing to these capacities, EEG remains a vital technique for detecting alterations in consciousness in clinical settings [57, 58]. There is currently strong evidence supporting the correlation between EEG patterns and anesthetic-induced unconsciousness [38, 56, 59-63]. Anesthetics generally decrease EEG power in higher frequency ranges and increase EEG power in lower frequencies [62]. However, different anesthetics exhibit distinct EEG characteristics owing to their respective molecular targets [12, 59] (Fig. **3**). Propofol, the most commonly used anesthetic, shifts the EEG into the alpha range (8-12 Hz), with a transition of synchronous alpha activity from the occipital lobes in the awake condition to the frontal lobes, a phenomenon referred to as anteriorization [57, 59, 62, 64] (Fig. **3A**). Along with this change, an increase in slow oscillations (<1 Hz) power has been identified [65]. The EEG responses to volatile anesthetics share similarities with those observed with propofol (Fig. **3B**). It should be noted that EEG exhibits age-dependent variability following volatile anesthetics administration. For instance, frontal alpha coherence was absent in infants younger than 1 year old, it became coherent at around 10 months old and it persisted to be prominent in adult age [58, 66]. Ketamine, a dissociative anesthetic, forms a unique gamma-burst pattern in EEG, characterized by alternating oscillations of gamma and delta waves, with a dose-dependent decrease in alpha connectivity from anterior to posterior regions during ketamine anesthesia [57, 67] (Fig. **3B**). Phase-amplitude coupling (PAC), where the amplitude of a higher frequency band is modulated by the phase of a lower frequency band, has been proposed as a neural mechanism for coordinating information processing across brain regions [68, 69]. During wakefulness, slow phase and alpha amplitude coupling is observed in parietal EEG, but not in frontal EEG [53]. Under the influence of multiple anesthetics, this PAC involving slow and alpha oscillations in parietal EEG weakens during the transition to unconsciousness [63, 70, 71]. It has been demonstrated that the posterior areas connect with thalamic nuclei delivering sensory information and communicate directly with the sensory cortex, while the prefrontal cortex connects with thalamic nuclei involved in cognition and communicates indirectly with the sensory cortex *via* posterior areas [72]. With an alteration in normal anterior-posterior connectivity, anesthetics dissociate sensory and cognitive processes by disrupting long-range connections to higher-order cortices, ultimately leading to a loss of consciousness. The causal relationship reflects a directed functional connection in the brain. Moreover, network studies focused on revealing the causal interactions between the frontal cortex and the parietal cortex based on EEG data. Ku *et al.* [73] employed eight EEG channels to assess the directional flow of information in the frontoparietal system during general anesthesia. They successfully demonstrated that the disruption of top-down feedback connectivity (frontal to parietal) is a common neurophysiological correlate of general anesthesia across two anesthetic classes (propofol and sevoflurane) and two analytical measures (evolutionary map approach and symbolic transfer entropy). Boly *et al.* [74] analyzed 256-electrode, high-density EEG recordings with dynamic causal modeling (DCM). The primary goal of DCM is to identify and quantify how neuronal activity in one region influences activity in another over time, particularly in response to propofol administration in this case. Their DCM findings suggested a selective impairment of backward connections in the context of preserved bottom-up forward connectivity (parietal to frontal) during propofol-induced loss of consciousness. It has been suggested that feedforward projections represent incoming sensory data, whereas feedback projections play a modulating role in the selection and contextual interpretation of information [75]. Combined with their roles, it suggests that general anesthesia suppresses higher-order cognitive processing while preserving lower-order sensory processing. The selective attenuation of feedback neural activity serves as a neural basis for the loss of consciousness induced by general anesthetics.

**Fig. (4):**
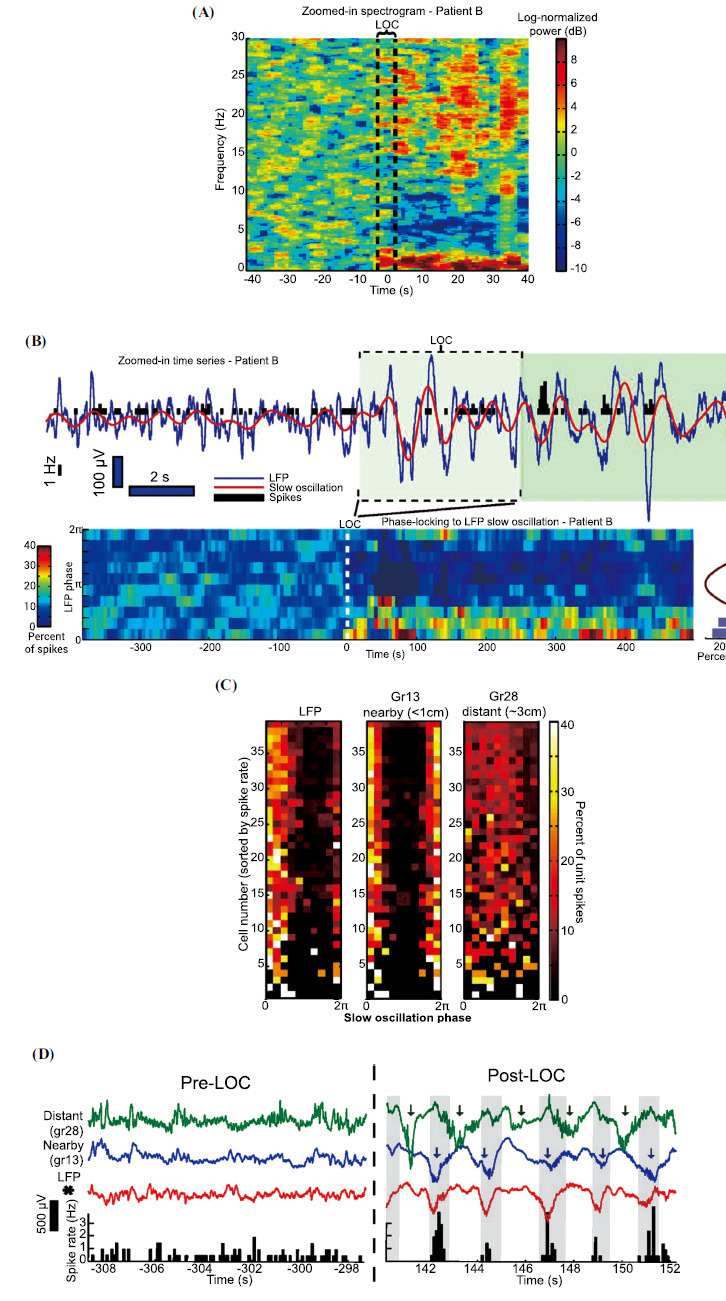
Slow oscillations emerge when entering the unconscious state induced by propofol and spikes become phase-coupled to the slow oscillation. (**A**) a representative spectrogram from a patient. The abrupt power increase after loss of consciousness (LOC) is specific to the slow oscillation. (**B**) Spikes were phase-coupled to the slow oscillation at LOC. (**C** and **D**) After LOC, slow oscillations are asynchronous, and distant cortices frequently are at a suppressed phase. Adapted from Lewis *et al*. [65].

Simultaneously recording single units, local field potentials (LFP), and EEG enables the detection of multiscale neural dynamics, encompassing ensembles of single neurons and oscillatory dynamics across distributed brain sites. Analyzing neural data from the human cortex alongside behavioral data from tasks, Lewis *et al.* [65] found slow oscillations in the LFP emerge when entering the unconscious state by propofol (Fig. **4A**), with neurons spiking only within specific phase windows of localized slow oscillations and remaining silent otherwise (Fig. **4B**). Furthermore, slow oscillations across the cortex become desynchronized, disrupting functional connectivity between distant cortical regions (Figs. **4C** and **4D**). These findings revealed that during unconsciousness, the brain enters a new state characterized by neuronal networks coupled to localized slow oscillations, leading to temporal and spatial functional fragmentation that disrupts cortical information integration.

Just as we discussed above, various electrophysiology and fMRI studies have proposed that, despite anesthetics targeting distinct molecular sites, they commonly produce unconsciousness through a decrease in fronto-parietal functional connectivity [38, 76-78]. However, Ma *et al.* [79] reported an unexpected finding that propofol increased long-range functional coupling of neural activity across a structurally connected fronto-parietal circuit. In detail, using a published monkey electrocorticography dataset, the researchers examined interactions within the cortical oculomotor circuit—a robust reciprocal anatomical projection from the frontal eye field in the frontal cortex to the lateral intraparietal area in the parietal cortex—during propofol anesthesia. Through coherence and inter-area PAC analyses, they observed similar increases in connectivity between other densely connected regions, such as the visual cortical areas V1 and V2, which also project to the frontal eye field and lateral intraparietal area. This suggested that the presence of dense direct projections, rather than specific analytic techniques, might account for the discrepancy. Another possible explanation for Ma *et al*.’s result is that in previous studies, EEG signals may not have been optimally sampled, as electrode grids were often placed randomly. Unlike Ma *et al.*, who directly compared functional coupling to anatomical projection, previous researchers preferred considering the distance between two areas in Euclidean terms rather than synaptic distance. Even though Euclidean distance between electrode sites typically correlates with synaptic distance between neuronal populations, this relationship can be disrupted by long-range projections, leading to potentially diminished functional connectivity maps [79]. As Mashour points out, increased functional connectivity does not necessarily imply an increase in overall information exchange between the frontal and parietal cortices. By isolating circuits or limiting their functional repertoire, high functional connectivity can, paradoxically, reflect a decrease in information transfer [80]. Notably, functional connectivity describes temporal correlations between spatially distant neurophysiological events and does not necessarily arise from anatomical connectivity [81]. In this case, increased fronto-parietal functional connectivity during anesthesia might indicate enhanced synaptic transmission *via* the oculomotor circuit, while reduced functional connectivity observed in prior studies might result from the suppression of an intermediate area when cortical areas lack dense direct projections between them. For instance, Warnaby and colleagues found a decrease in fronto-parietal functional connectivity between the dorsolateral prefrontal cortex and inferior parietal lobule, alongside fMRI evidence for suppression of the dorsal anterior insula as an intermediate area [55]. Ma *et al*.'s work highlights the need for a more careful reconsideration of the role of fronto-parietal corticocortical interactions and underscores the importance of studying the projection anatomy within fronto-parietal networks in the context of anesthetic-induced unconsciousness.

**Fig. (5):**
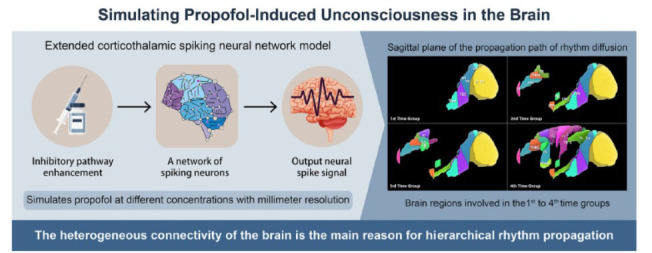
Simulate the altered states of consciousness induced by propofol. Adapted from Zhang *et al*. [84].

With the rapid advancements in artificial intelligence, faster and more accurate tools are now available for analyzing changes in interactions between brain regions during the gradual disappearance of consciousness. Propofol completes an arm-brain cycle in mere seconds, rapidly inducing anesthesia in surgical patients [60, 82, 83]. Therefore, traditional means of commonly used clinical observation are insufficient to accurately capture the rapid, complex changes in signaling processing across various brain regions during this brief timeframe. For example, fMRI does not directly track the distribution of gradient rhythms of interactions between brain regions as consciousness fades. Addressing this challenge, Yu’s team [84] constructed a biologically constrained spiking neural network model with millisecond resolution by applying computational neuroscience. The model could simulate the altered states of consciousness induced by varying propofol concentrations (Fig. **5**). During the gradual deepening of anesthesia, they discovered, for the first time, four gradients of rhythm diffusion in time, as well as the hierarchical spatial distribution of dominant brain regions progressing from the occipital lobe to the frontal lobe and then to the whole brain. This innovative integration of neuroscience data with computational modeling and simulation of brain neurons has uncovered the complex and hierarchical interactions between different brain regions. This research specifically focuses on the anteriorization of alpha rhythms during the loss of consciousness induced by anesthesia, offering new insights for the study of consciousness.This model could lead to the development of next-generation monitoring systems, it will provide a more accurate and objective way to monitor anesthesia depth by offering more detailed insights into brain dynamics during anesthesia. In-depth integration of brain science, computational neuroscience, and artificial intelligence holds transformative potential for tackling foundational scientific questions about consciousness. By advancing interdisciplinary research in computational modeling, we can make significant strides toward addressing these challenges, with implications for both theoretical understanding and practical applications in brain-inspired intelligence.

## 5 THE CORTICO-SUBCORTICAL MECHANISM UNDERLYING GENERAL ANESTHESIA

Maintaining normal levels of consciousness and the contents of consciousness requires the collaborative involvement of both cortical and subcortical regions. Subcortical structures, particularly the thalamus, play a crucial role in relaying sensory information to the cortex, where it undergoes complex processing and integration. Once the cortical regions process and synthesize the incoming data, feedback signals are sent back to the thalamus, creating a bidirectional communication loop. This continuous exchange between the subcortex and cortex facilitates the integration of sensory inputs, attentional processes, and higher cognitive functions. Through this dynamic interaction, the brain constructs a coherent representation of the environment, which forms the basis for conscious experience [85].

**Fig. (6):**
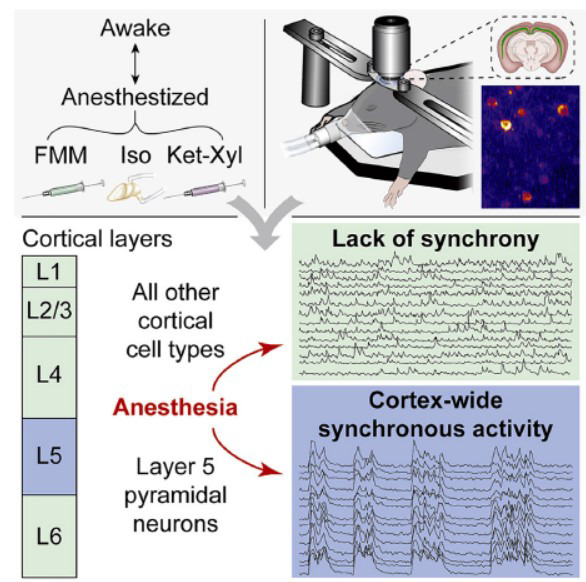
General anesthesia globally synchronizes activity selectively in layer 5 cortical pyramidal neurons. Adapted from Bharioke *et al*. [16].

Clinical and experimental results suggest that general anesthetics disrupt neural activity across multiple brain sites to induce loss of consciousness, including the sleep-wake system [4, 11, 86, 87]. Anesthetics likely degrade the contents of consciousness primarily by acting on the cortex while reducing arousal by targeting subcortical areas. Early research predominantly focused on the role of subcortical nuclei, suggesting that anesthetics inhibit these subcortical structures and reduce the sensory input to the cortex, thereby inducing unconsciousness [11]. However, this perspective overlooks the critical “top-down” control exerted by the cortex on subcortical structures. Numerous recently published studies have demonstrated that anesthetic-induced unconsciousness relies heavily on “top-down” mechanisms or the direct effects of anesthetics on cortical neurons [61, 88]. General anesthetics, such as propofol, isoflurane, and ketamine, have been shown to directly inhibit the spontaneous activity of cortical neurons by binding with cortical receptors. General anesthetics have been reported to suppress cortical neuronal activity by 40%-50% at doses sufficient to induce loss of righting reflex (LORR) in animals [87]. It is worth mentioning that ketamine could activate certain cortical areas, the limbic system, and the hippocampus due to its disinhibitory properties [87]. This activation may lead to changes in the spontaneous activity of cortical neurons, which can affect consciousness. Additionally, Bharioke *et al.* [16] found that different general anesthetics consistently synchronized activity selectively in layer 5 cortical pyramidal neurons among the whole brain and that transitions in consciousness during anesthesia closely correspond with the onset and cessation of this synchronous activity across layer 5 neurons (Fig. **6**).

**Fig. (7):**
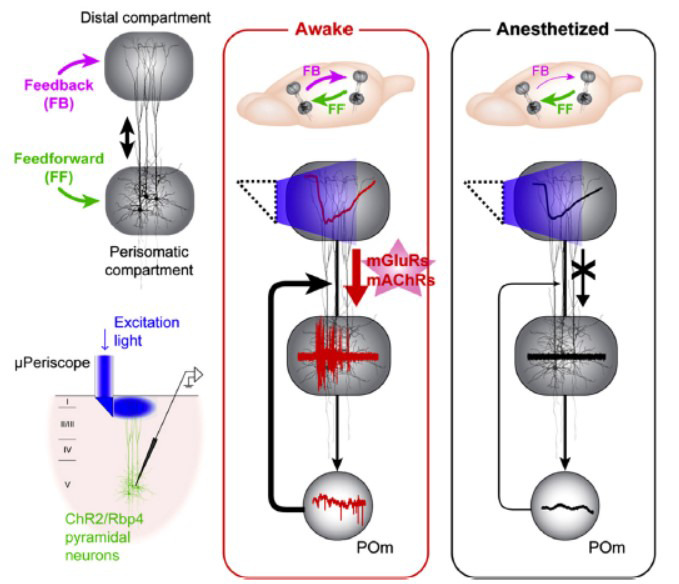
General anesthetics decoupled the signal transmission from distal dendrites to the soma in pyramidal neurons. Adapted from Suzuki and Larkum [89].

More specifically, dendro-somatic decoupling in cortical pyramidal neurons may represent a cellular mechanism by which anesthesia disrupts cortico-cortical connectivity and, consequently, suppresses consciousness [89, 90]. Suzuki *et al.* [89] optogenetically stimulated the distal compartments of these neurons and measured activity propagation to the perisomatic compartment under 1% isoflurane, ketamine/xylazine, or urethane anesthesia. They observed that these anesthetic agents significantly decoupled the signal transmission from distal dendrites to the soma in layer 5 neurons, leading to diminished cortical column output because of limited distal dendritic input. The overall effect was widespread decoupling of feedback connections throughout the cortex [16] (Fig. **7**). Nonetheless, further research is needed to elucidate the neural circuit mechanisms underlying anesthesia-induced functional disconnection within fronto-parietal networks.

The frontal and parietal cortices serve distinct roles, and their interactions may provide conditions sufficient for the emergence of conscious experience [7, 91-93]. Pal *et al.* [19] demonstrated that while cholinergic and noradrenergic stimulation of prefrontal and parietal cortices can activate the cortex, only cholinergic stimulation of the prefrontal cortex restored consciousness and reversed the anesthetized state. Their work highlighted the critical role of the prefrontal cortex in modulating the level of consciousness, although the parietal cortex's precise contribution to anesthetic-induced unconsciousness remains ambiguous. Research on neural correlates of consciousness has traditionally focused on two aspects: the contents of conscious experience and the level of consciousness [94]. As a result, it would be rash to exclude the potential role of additional brain areas, like the parietal cortex, in restoring the full spectrum of consciousness, given its known involvement in conscious content. Nowadays, it is increasingly recognized that the levels and contents of consciousness cannot be completely dissociated. The fronto-parietal network may serve as a hub for regulating both the level and content of consciousness, as disruption in this neural network is a remarkable feature of anesthesia-induced shifts in conscious state.

## 6 MOLECULAR MECHANISMS UNDERLYING DISRUPTION OF FRONTO-PARIETAL CORTICAL CONNECTIVITY

Taking a step further, at the molecular level, commonly used anesthetic agents exert their effects *via* binding to corresponding protein receptors, thereby altering neuronal excitability and synaptic transmission [12]. These molecular mechanisms directly or indirectly disrupt fronto-parietal cortical connectivity, which is critical for maintaining conscious awareness and higher-order cognitive functions. Propofol, for instance, potentiates inhibitory neurotransmission primarily through binding to synaptic and extrasynaptic γ-aminobutyric acid type A (GABA_A_) receptors, leading to neuronal hyperpolarization and inhibition. Besides, propofol interacts with hyperpolarization-activated cyclic nucleotide-gated (HCN) channels, further augmenting its anesthetic properties in the brain [12]. The hyperpolarization of neurons in frontal and parietal cortices reduces the synchrony of oscillatory activity (*e.g*., alpha and gamma bands), which is essential for long-range communication between these regions. Propofol’s suppression of HCN channels in thalamocortical relay neurons disrupts thalamic input to the frontal and parietal cortices, impairing their functional coupling [37]. Sevoflurane, another widely used anesthetic, is also known for its ability to enhance the inhibitory actions of GABA_A_ receptors. Additionally, it may modulate two-pore domain potassium channels (K_2p_) and voltage-gated sodium channels, contributing to its anesthetic effects on neurons [59]. Enhanced GABAergic inhibition suppresses pyramidal neuron activity in the frontal cortex, diminishing top-down signaling to the parietal cortex. K_2_p channel activation reduces neuronal excitability in parietal associative regions, weakening bottom-up sensory processing [52]. In contrast, ketamine functions mainly by blocking excitatory N-methyl-D-aspartate (NMDA) glutamate receptors. Once NMDA receptors in interneurons are inhibited, the downstream excitatory neurons become disinhibited or more active [90]. The gamma oscillation induced by ketamine may be the consequence of ketamine preferentially blocking cortical interneurons, which raises pyramidal neuronal activity and creates aberrant synchronization that disrupts normal fronto-parietal interactions. Consciousness is lost when the NMDA receptors on the excitatory pyramidal neurons are inhibited as ketamine dosage increases. All three anesthetics alter the balance between inhibitory (GABAergic) and excitatory (glutamatergic) neurotransmission. Propofol and sevoflurane amplify inhibition, while ketamine suppresses excitation [95-97]. Excessive GABAergic inhibition (propofol/ sevoflurane) or glutamatergic dysregulation (ketamine) destabilizes the excitation-inhibition balance required for coherent fronto-parietal network activity. This imbalance reduces the efficiency of information transfer and integration between frontal executive regions and parietal sensory hubs.

In summary, anesthetic agents reconfigure cortical neurotransmitter networks by targeting specific receptors, thereby altering neuronal excitability and synaptic transmission, finally arriving at the anesthetic state [98-100]. Future studies should explore how receptor-specific manipulations (*e.g*., selective GABA_A_ or NMDA modulators) differentially affect fronto-parietal networks to refine anesthesia monitoring and consciousness research.

## 7 CLINICAL IMPLICATIONS AND FUTURE DIRECTIONS

All anesthetics seem to ultimately interrupt the integration of information across a broad set of higher-order cortical regions, especially the “cross-talks” between the frontal and parietal cortices [101]. It is promising that several clinical implications will arise from the efforts to dissect mechanisms of general anesthesia-associated fronto-parietal cortical connectivity breakdown. For instance, a deeper understanding of how anesthesia affects fronto-parietal communication could enable the development of more accurate anesthetic depth monitoring systems. These systems would allow anesthesiologists to tailor frontal-parietal connectivity in real time, minimizing the risks associated with over-sedation or under-sedation, and thereby enhancing patient safety during surgeries. Furthermore, the insights gained from studying the breakdown of fronto-parietal connectivity could pave the way for the development of new anesthetic agents with more targeted and selective actions. Such agents may target special fronto-parietal circuits to potentially minimize undesirable side effects, such as cognitive dysfunction or long-term post-anesthesia delirium, while maintaining effective unconsciousness.

At present, the exact mechanisms by which anesthetics lead to the functional disconnection of fronto-parietal networks remain unclear. A simple hypothesis is that anesthetic agents inhibit the activity of direct neural projections between the parietal and frontal lobes. However, the anatomy of fronto-parietal functional network is likely more complex. Future research should focus on specific molecular receptor targets, types of neurons, and neurotransmitters involved, as well as how anesthetic agents alter neural firing and synaptic transmission. The research could elucidate how these factors eventually contribute to the temporal decoupling of parietal and frontal signals.

As another topic to be addressed by future studies, it would be worthwhile to study the causality between altered activities of the fronto-parietal network and the unconscious state. Combining neuromodulatory tools with non-invasive functional connectivity assessment methods, such as fMRI and EEG, can solve this issue. This methodology enables the direct assessment of changes in conscious level and content resulting from the modulation of functional interactions [102-104]. Although it is challenging to integrate neuromodulatory techniques in clinical settings, the development of optogenetics and chemogenetics in animal models, especially non-human primate models, progressively allows the regulation of activity in specifically targeted neuron populations with spatiotemporal specificity. This advancement is of great importance for the field [105].

## CONCLUSION

**Fig. (8):**
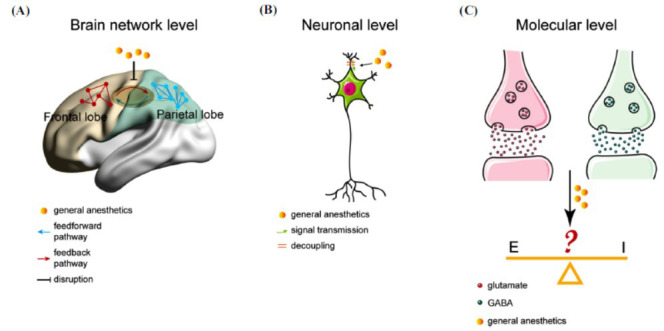
Simplified multiscale scheme of the neurobiological mechanisms of anesthesia-induced unconsciousness, focused on the disruption of fronto-parietal cortical connection. (**A**) Brain network mechanism, (**B**) Neuronal mechanism, (**C**) Molecular mechanism.

The transition from consciousness to unconsciousness during anesthesia remains one of the most intricate phenomena in neuroscience, a process that has not been fully understood despite over one and a half centuries of research. A long-standing theory that emphasizes the critical role of interrupted fronto-parietal cortical connectivity under general anesthesia continues to hold significant weight in current discussions on the neurobiological basis of anesthesia. This theory suggests that the integrity of connections between the frontal and parietal regions of the brain is essential for the integration of information that allows for conscious awareness; anesthetics disrupt this communication to induce unconsciousness. Numerous functional imaging and electrophysiological studies have consistently demonstrated this breakdown in the functional connectivity between the frontal and parietal cortices during general anesthesia, confirming the pivotal role these brain regions play in maintaining conscious states. More specifically, anesthetic agents induced loss of consciousness coupled with decoupling of dendrites from somas (dendro-somatic decoupling), as well as significant reconfigurations in the network of cortical neurotransmitters (Fig. **8**). The reorganization of neurotransmitter systems during anesthesia likely affects both local and long-range communication within the cerebral cortex, impairing the integration of information that is essential for maintaining regular brain function. These changes at the cellular level may provide a mechanistic explanation for the global dynamic shift that occurs in the brain during anesthesia, ultimately leading to the loss of conscious awareness.

In the years to come, advancing our understanding of the cellular and network-level mechanisms that underlie anesthesia-induced unconsciousness will not only provide valuable insights into the process of anesthesia itself but also illuminate broader principles of other vital brain functions, such as sleep, arousal, and consciousness. In this article, by reviewing a large number of important studies, we propose that various anesthetics cause loss of consciousness by interrupting the connections between the parietal and frontal lobes, and further elaborate on the specific neural circuit mechanisms involved. Ultimately, this research offers profound implications for both clinical anesthesia and our understanding of the fundamental neurological basis of conscious experience.

## AUTHORS’ CONTRIBUTIONS

The authors confirm their contribution to the paper as follows: study conception and design: YYH, XC; data analysis and interpretation: WC, SS; investigation: KW, JK, YXH; draft manuscript: YD, SL. All authors reviewed the results and approved the final version of the manuscript.

## Figures and Tables

**Table 1:** Electroencephalographic and fMRI Studies in the last decade (2014—2024) that found frontal-parietal connectivity altered during general anesthesia.

**Study**	**Participants**	**Anesthetics**	**Technique**	**Results**
Liang *et al.* (2024) [38]	72 surgical patients	Propofol, dexmedetomidine, or ketamine	EEG-derived permutation cross-mutual information	Decreased frontal-parietal and parietal-occipital connectivity
Chen *et al.* (2022) [47]	14 patients	Propofol	EEG-derived weighted phase lag index and directed phase transfer entropy	Decreased posterior-to-anterior (feedforward) directed connectivity
Zhang *et al.* (2019) [48]	16 monkeys	Isoflurane	fMRI bold correlation	Decreased parietal and temporal/prefrontal cortex connections Increased connectivity in default mode network
Kim *et al.* (2024) [49]	52 surgical patients	Remimazolam and propofol	EEG normalized symbolic transfer entropy	Greater decrease in frontoparietal feedback connectivity than the decrease in feedforward connectivity
Sanders *et al.* (2018) [50]	8 patients	Propofol	EEG dynamic causal modeling	Impairments in feedforward and feedback connections between parietal and occipital cortex
Lv *et al.* (2016) [51]	14 monkeys	Isoflurane	fMRI bold correlation	Reduced default mode network and frontal-parietal connectivity
Maksimow *et al.* (2014) [52]	10 healthy men	Propofol	EEG Granger causality	Decrease in occipital-to-frontal connectivity Increase in frontal-to-occipital connectivity
Blain-Moraes *et al.* (2015) [53]	10 healthy volunteers	Sevoflurane	EEG-derived phase lag index	Disruption of anterior-posterior interaction
Vlisides *et al.* (2018) [54]	15 healthy volunteers	Ketamine	EEG correlation	Functional lesions involving anterior-posterior junction

## LIST OF ABBREVIATIONS

DCM: Dynamic Causal Modeling
DTI: Diffusion Tensor Imaging
EEG: Electroencephalography
IIT: Integrated Information Theory
LFP: Local Field Potentials
PAC: Phase-amplitude Coupling
SLF: Superior Longitudinal Fasciculus
